# Thrombus Compositional Balance Is Associated with Complete Reperfusion After Mechanical Thrombectomy

**DOI:** 10.3390/jcm15208061

**Published:** 2026-10-17

**Authors:** Jang-Hyun Baek, Hyo Suk Nam, JoonNyung Heo, Il Kwon, Byung Moon Kim, Dong Joon Kim, Young Dae Kim

**Affiliations:** 1 Department of Neurology, Kangbuk Samsung Hospital, Sungkyunkwan University School of Medicine, Seoul 03181, Republic of Korea; 2 Department of Neurology, Severance Stroke Center, Severance Hospital, Yonsei University College of Medicine, Seoul 03722, Republic of Korea; 3 Integrative Research Center for Cerebrovascular and Cardiovascular Diseases, Yonsei University College of Medicine, Seoul 03722, Republic of Korea; 4 Division of Interventional Neuroradiology, Department of Radiology, Severance Stroke Center, Severance Hospital, Yonsei University College of Medicine, Seoul 03722, Republic of Korea

**Keywords:** mechanical thrombectomy, thrombus composition, compositional data analysis, complete reperfusion

## Abstract

**Background/Objectives:** Thrombus composition has been associated with the response to mechanical thrombectomy (MT), but previous studies have largely evaluated individual components separately or classified thrombi using component-rich categories. We investigated whether the relative compositional balance among red blood cells (RBCs), platelets, and fibrin was associated with endovascular outcomes after MT. **Methods:** We retrospectively analyzed 312 patients at a single center who underwent MT for acute intracranial large-vessel occlusion between 2017 and 2021 and had retrieved thrombi available for histological analysis. RBC, platelet, and fibrin proportions were quantified immunohistochemically. An isometric log-ratio transformation was used to derive a compositional balance contrasting RBCs with platelets and fibrin, with higher values indicating greater relative RBC predominance. Endovascular outcomes were compared across quartiles of compositional balance, and exploratory logistic regression analyses were performed to further characterize the observed association with complete reperfusion (modified Thrombolysis in Cerebral Infarction [mTICI] 3). **Results:** Complete reperfusion was achieved in 65.7% of patients. Rates were 56.4%, 62.8%, 73.1%, and 70.5% from the lowest to the highest quartile (Q1–Q4) of compositional balance (*p* for trend = 0.030). Compositional balance was not associated with successful reperfusion, first-pass effect, or the number of thrombectomy passes. Higher compositional balance was associated with greater odds of complete reperfusion both continuously (adjusted odds ratio [OR] per 1-unit increase, 1.31; 95% confidence interval [CI], 1.03–1.67; *p* = 0.027) and across increasing quartiles (adjusted OR per quartile increase, 1.41; 95% CI, 1.11–1.80; *p* = 0.005). The association remained consistent after excluding thrombi with a platelet proportion < 1%. **Conclusions:** In this exploratory analysis, greater relative RBC predominance over platelets and fibrin was associated with a higher likelihood of complete reperfusion after MT. The relative balance among thrombus components may provide additional insight into how thrombus composition relates to the completeness of endovascular reperfusion after MT.

## 1. Introduction

Mechanical thrombectomy (MT) is the standard treatment for acute ischemic stroke caused by large vessel occlusion, but procedural efficacy varies substantially among patients [1]. Because thrombectomy relies on mechanical interaction between the thrombus and retrieval devices, thrombus composition may influence endovascular outcomes [2,3,4]. Experimental and biomechanical studies have shown distinct mechanical properties according to thrombus composition, with red blood cell (RBC)-rich thrombi generally being softer and more deformable and platelet/fibrin-rich thrombi being stiffer and more resistant to deformation [2,4,5]. Clinical studies have accordingly reported associations of higher RBC or lower platelet/fibrin content with favorable reperfusion and procedural outcomes, although findings have not been consistent across studies [6,7,8,9,10].

Most previous studies, however, have evaluated RBCs, platelets, and fibrin as separate variables or classified thrombi as component-rich or component-poor using study-specific thresholds [2,11]. In this context, such approaches may not adequately capture the relative predominance of thrombus components. A compositional approach may therefore provide a more integrated representation of the balance among these components.

Accordingly, we applied compositional data analysis to characterize the relative predominance of RBCs over platelets and fibrin as an isometric log-ratio–derived compositional balance. We investigated the association between this RBC-to-platelet/fibrin balance and endovascular outcomes after MT, hypothesizing that greater relative RBC predominance would be associated with more favorable endovascular responses.

## 2. Materials and Methods

### 2.1. Study Population

We retrospectively analyzed consecutive patients with acute ischemic stroke due to intracranial large-vessel occlusion who underwent endovascular treatment (EVT) at a tertiary stroke center between 2017 and 2021. Treatment eligibility was determined according to contemporary clinical and imaging criteria. Patients eligible for intravenous thrombolysis received alteplase at a standard dose of 0.9 mg/kg within the approved time window in the absence of contraindications. Patients with intracranial atherosclerotic stenosis-related occlusion or other uncommon occlusion etiologies, including in-stent occlusion, arterial dissection, and moyamoya disease, were excluded. Among patients with eligible occlusion etiologies, those in whom no thrombus was retrieved were further excluded.

### 2.2. Mechanical Thrombectomy Procedure

MT was performed using a stent retriever or contact aspiration at the discretion of the treating neurointerventionalist. A stent retriever was used as the first-line device in most cases. For stent retriever thrombectomy, a microcatheter was advanced across the occluded segment over a microwire, and the stent retriever was deployed across the thrombus. For anterior circulation occlusions, an 8- or 9-F balloon guide catheter was routinely used. After deployment of the stent retriever, the balloon was inflated to achieve proximal flow arrest, and the stent retriever was withdrawn under continuous manual aspiration through the balloon guide catheter. For contact aspiration thrombectomy, a large-bore aspiration catheter was advanced to the proximal surface of the thrombus over a microcatheter and microwire. After engagement of the thrombus, aspiration was applied through the aspiration catheter, which was subsequently withdrawn while maintaining continuous aspiration. In patients with substantial vascular tortuosity, a distal access catheter was used to facilitate access to the target vessel. Concurrent contact aspiration with stent retriever thrombectomy was generally avoided and was reserved for selected cases in which standard approaches were unsuccessful. Thrombectomy was generally repeated with the goal of achieving complete reperfusion (modified Thrombolysis in Cerebral Infarction [mTICI] 3). Decisions regarding additional attempts, conversion to an alternative thrombectomy technique, or termination of the procedure were made by the treating neurointerventionalist based on technical feasibility and clinical considerations.

### 2.3. Thrombus Staining and Analysis

Retrieved thrombi were immediately fixed in 4% paraformaldehyde, paraffin-embedded, and sectioned at a thickness of 4 μm. For the present analysis, red blood cells (RBCs), platelets, and fibrin were identified immunohistochemically using antibodies against glycophorin A, CD42b, and fibrinogen, respectively. Immunoreactive signals were visualized using 3,3′-diaminobenzidine. Images of the stained thrombi were acquired using either a whole-slide scanner (Leica Biosystems, Richmond, IL, USA) or a Stereo Investigator Imaging system (MBF Bioscience, Williston, VT, USA). The acquired images were quantitatively analyzed using Automated Region-of-interest-based Image Analysis (ARIA) software [12]. Thrombus processing and image analysis were performed using an established laboratory workflow with routine quality-control review, independently of the specific aims and outcomes of the present study. The proportion of each thrombus component was calculated as the positively stained area divided by the corresponding total thrombus area and expressed as a percentage.

Given the established relevance of RBCs, platelets, and fibrin to the mechanical properties of thrombi and their response to thrombectomy [2,4,5], these three components were selected to define a mechanically relevant subcomposition for the present analysis. This subcomposition was not intended to exhaustively represent the entire biological composition of the thrombus, nor were the independently quantified component proportions assumed to constitute mutually exclusive spatial fractions. Rather, our compositional contrast of interest was the relative predominance of RBCs over platelets and fibrin. To characterize this relative predominance, an isometric log-ratio (ILR) balance was calculated according to the following equation [13,14,15]:

$$\sqrt{\frac{2}{3}}\ln\left(\frac{\mathrm{RBC}\%}{\sqrt{\mathrm{Platelet}\%\times \mathrm{Fibrin}\%}}\right)$$

where *ln* denotes the natural logarithm and RBC%, Platelet%, and Fibrin% represent the immunohistochemically quantified proportions of the respective components. The three proportions were not explicitly renormalized to sum to 1 before transformation. The resulting coordinate was termed compositional balance and was used as the principal compositional variable in subsequent analyses. Higher values indicate greater relative RBC predominance over platelets and fibrin, whereas lower values indicate the opposite.

### 2.4. Endovascular Outcomes

Endovascular outcomes included successful reperfusion, complete reperfusion, first-pass effect, and the number of thrombectomy passes. The degree of reperfusion was assessed using the mTICI scale. Successful reperfusion was defined as a final mTICI grade of 2b or 3, whereas complete reperfusion was defined as a final mTICI grade of 3. First-pass effect was defined as achieving mTICI 2c or 3 after a single thrombectomy pass, sustained without additional intervention [16]. The total number of thrombectomy passes was recorded, and the requirement for three or more passes was additionally evaluated as a categorical outcome. The final reperfusion outcomes were determined by consensus during regular meetings involving stroke neurologists and neurointerventionalists. These assessments were performed independently of the aims of the present study, and thrombus histological findings were not considered during angiographic outcome adjudication.

### 2.5. Statistical Analysis

First, to examine the association between thrombus composition and endovascular outcomes, patients were divided into quartiles according to compositional balance. Continuous outcomes were compared across quartiles using one-way analysis of variance or the Kruskal–Wallis test, as appropriate, and categorical outcomes were compared using the Pearson chi-square test or Fisher’s exact test. For binary endovascular outcomes, trends across increasing quartiles of compositional balance were evaluated by treating quartile assignment as an ordinal variable in logistic regression.

Second, in this exploratory analysis, endovascular outcomes showing an overall difference or trend across compositional-balance quartiles were further evaluated using regression analyses to characterize the observed associations. Because no single parameterization has been established to best represent the relationship between thrombus composition and endovascular outcomes, compositional balance was examined using complementary parameterizations: as a continuous variable, as categorical quartiles with the lowest quartile serving as the reference group, and as an ordinal quartile variable to summarize the overall association across ordered compositional-balance categories. Both univariable and multivariable regression analyses were performed. For multivariable adjustment, stroke etiology, occlusion site, intravenous alteplase administration, tandem occlusion, and first-line thrombectomy strategy were included as clinically relevant variables based on their potential relationships with thrombus composition and endovascular reperfusion, irrespective of their univariable associations. In addition, age, hypertension, diabetes mellitus, dyslipidemia, and onset-to-puncture time, which were associated with complete reperfusion at *p* < 0.10 in univariable analyses, were included in the models. Effect estimates are presented with 95% confidence intervals (CIs). In additional exploratory analyses, potential effect modification was assessed by testing interaction terms between compositional balance and first-line thrombectomy strategy, occlusion site, tandem occlusion, intravenous alteplase administration, stroke etiology, and treatment year. *p* values for interaction were obtained using likelihood-ratio tests comparing models with and without the corresponding interaction term.

Third, because very low platelet proportions may disproportionately increase the ILR-derived compositional balance independent of greater RBC predominance, a sensitivity analysis was performed after excluding thrombi with a platelet proportion < 1%. Compositional-balance quartiles were redefined within the remaining cohort, and the quartile-based comparisons and regression analyses were repeated using the same analytic approach as in the primary analysis.

All statistical analyses were performed using R software (version 4.5.1; R Foundation for Statistical Computing, Vienna, Austria). R code for calculating the compositional balance and fitting the principal multivariable regression model is provided in the Supplementary Methods. A two-sided *p* value < 0.05 was considered statistically significant.

## 3. Results

**Figure 1:**
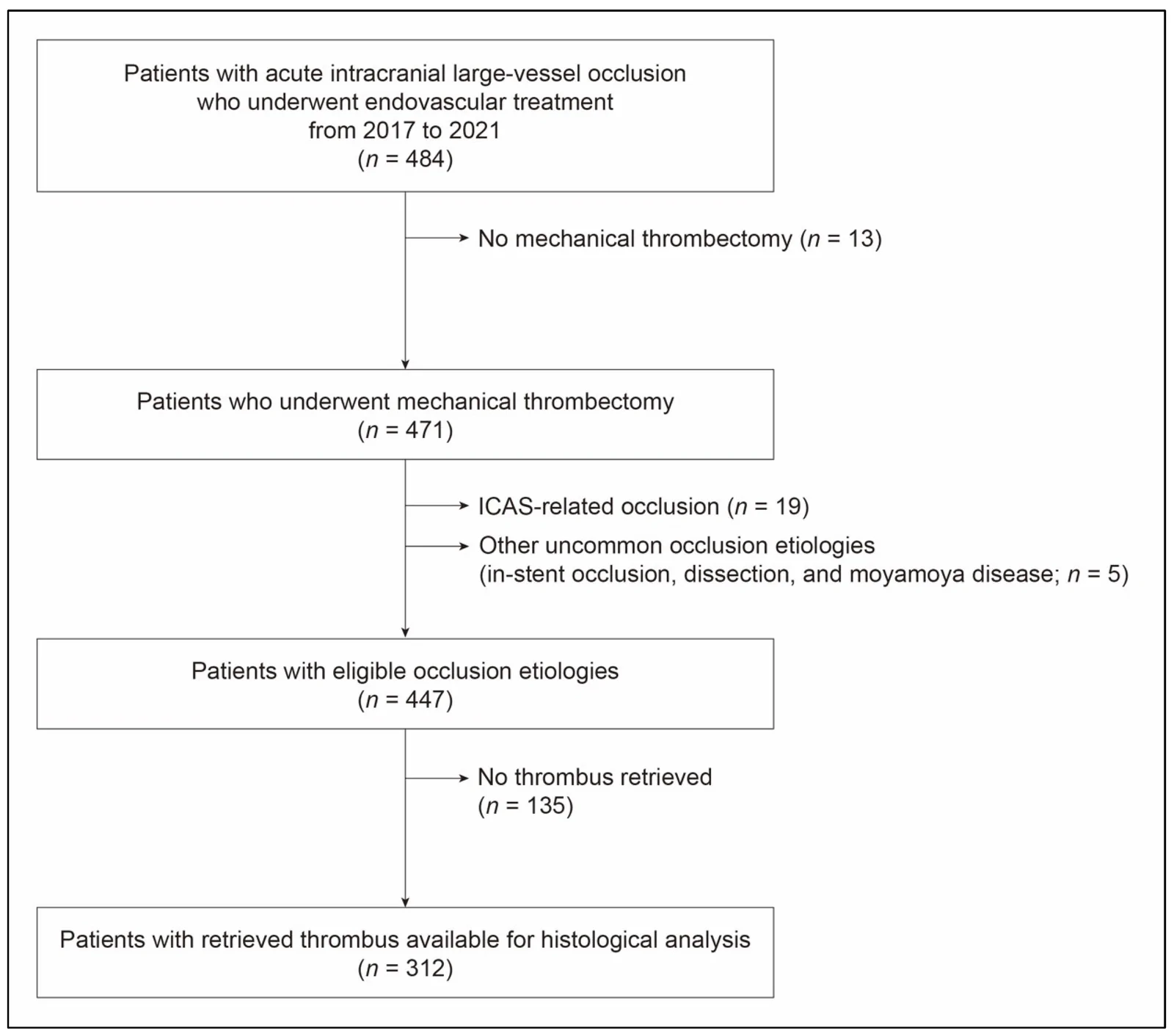
Flow of patient selection. ICAS, intracranial atherosclerotic stenosis.

Among 484 patients with acute intracranial large-vessel occlusion who underwent EVT during the study period, 312 patients with retrieved thrombi available for histological analysis were included in the final study population (Figure 1). Among the excluded patients, 13 did not undergo mechanical thrombectomy; their EVTs included intra-arterial thrombolysis, mechanical disruption, stenting, percutaneous transluminal angioplasty, and intra-arterial glycoprotein IIb/IIIa inhibitor administration. The median initial NIHSS score was 14 (IQR, 10–19), and the median onset-to-puncture time was 249 min (IQR, 139–545). The most common occlusion sites were M1 (36.5%), ICA (27.9%), and M2 (24.4%) (Table 1). Histological analysis showed median proportions of 36.6% (IQR, 22.0–47.4) for RBCs, 9.1% (IQR, 3.8–17.9) for platelets, and 32.4% (IQR, 21.5–47.4) for fibrin. The median compositional balance, representing the RBC-to-platelet/fibrin balance, was 0.62 (IQR, 0.08–1.04).

### 3.1. Endovascular Outcomes According to Compositional Balance

For analyses according to compositional balance, patients were divided into quartiles with the following boundaries: Q1, ≤0.084; Q2, >0.084 to ≤0.624; Q3, >0.624 to ≤1.041; and Q4, >1.041. Baseline and procedural characteristics stratified by compositional-balance quartiles are provided in Supplementary Table S1. Successful reperfusion was achieved in 291 patients (93.3%) and did not differ across compositional-balance quartiles (overall *p* = 0.905; *p* for trend = 0.920). No significant differences across quartiles or trends were observed for first-pass effect (overall *p* = 0.777; *p* for trend = 0.571), number of thrombectomy passes (*p* = 0.853), or the proportion requiring three or more passes (overall *p* = 0.920; *p* for trend = 0.824) (Table 2).

Complete reperfusion was achieved in 205 patients (65.7%). The rates of complete reperfusion were 56.4%, 62.8%, 73.1%, and 70.5% from Q1 through Q4, respectively. Although the overall comparison across the four quartiles was not statistically significant (*p* = 0.114), there was a significant trend toward higher rates of complete reperfusion across increasing quartiles of compositional balance (*p* for trend = 0.030) (Table 2).

### 3.2. Association Between Compositional Balance and Complete Reperfusion

In multivariable logistic regression analysis, higher compositional balance was associated with greater odds of complete reperfusion when analyzed as a continuous variable (adjusted odds ratio [aOR] per 1-unit increase, 1.31; 95% CI, 1.03–1.67; *p* = 0.027) (Table 3 and Supplementary Table S2). When compositional balance was analyzed as quartiles, patients in Q3 (aOR, 2.59; 95% CI, 1.22–5.49; *p* = 0.013) and Q4 (aOR, 2.44; 95% CI, 1.15–5.17; *p* = 0.020) had higher odds of complete reperfusion than those in Q1, whereas no significant difference was observed between Q2 and Q1 (aOR, 1.11; 95% CI, 0.55–2.27; *p* = 0.770). In the ordinal analysis, increasing compositional-balance quartiles were associated with higher odds of complete reperfusion (aOR per quartile increase, 1.41; 95% CI, 1.11–1.80; *p* = 0.005).

In additional interaction analyses, the association between compositional balance and complete reperfusion did not differ significantly according to first-line thrombectomy strategy, occlusion site, tandem occlusion, intravenous alteplase administration, stroke etiology, or treatment year (all *p* for interaction > 0.05).

### 3.3. Sensitivity Analysis

To assess the potential influence of near-zero platelet proportions on the log-ratio-derived compositional balance, a sensitivity analysis was performed after excluding 13 patients with platelet proportions < 1%. Compositional-balance quartiles were redefined among the remaining 299 patients. The association between compositional balance and complete reperfusion was materially unchanged after this exclusion. The adjusted effect estimate for compositional balance as a continuous variable was similar to that in the primary cohort (aOR per 1-unit increase, 1.32; 95% CI, 1.03–1.68; *p* = 0.030), and increasing compositional-balance quartiles were similarly associated with higher odds of complete reperfusion (aOR per quartile increase, 1.49; 95% CI, 1.17–1.91; *p* = 0.002) (Table 3).

**Figure 2:**
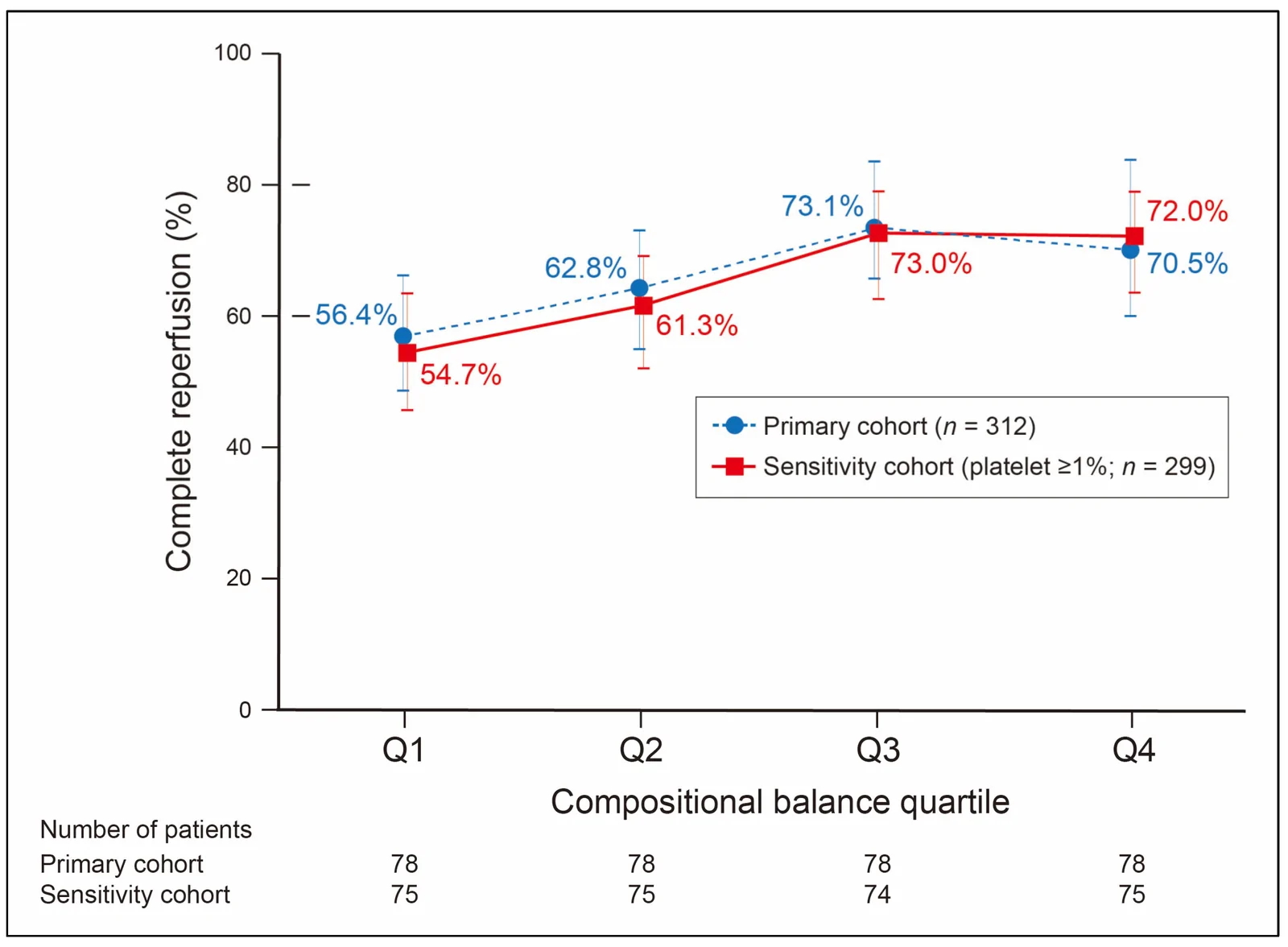
Complete reperfusion according to compositional balance in the primary and sensitivity cohorts. Points represent the proportions of patients achieving complete reperfusion (mTICI 3) across quartiles of compositional balance, and error bars indicate 95% confidence intervals. Complete reperfusion rates increased from Q1 through Q3, with little further change in Q4, in both the primary and sensitivity cohorts. In the primary cohort, quartile boundaries were Q1, ≤0.084; Q2, >0.084 to ≤0.624; Q3, >0.624 to ≤1.041; and Q4, >1.041. In the sensitivity analysis, 13 patients with a platelet proportion <1% were excluded, and quartiles were redefined within the remaining 299 patients as Q1, ≤0.041; Q2, >0.041 to ≤0.596; Q3, >0.596 to ≤1.006; and Q4, >1.006. Compositional balance represents the RBC-to-platelet/fibrin balance derived using an isometric log-ratio transformation. mTICI, modified Thrombolysis in Cerebral Infarction.

Complete reperfusion rates in the sensitivity cohort were 54.7%, 61.3%, 73.0%, and 72.0% from Q1 through Q4, respectively, consistent with the pattern observed in the primary cohort (*p* for trend = 0.025) (Figure 2).

## 4. Discussion

In this study, a higher thrombus compositional balance, representing greater relative RBC predominance over platelets and fibrin, was associated with an increased likelihood of complete reperfusion after MT. This association was observed when compositional balance was analyzed continuously and remained evident in the ordinal quartile analysis after adjustment for potential confounders. In contrast, compositional balance was not associated with successful reperfusion, first-pass effect, or the number of thrombectomy passes. These exploratory findings raise the possibility that the relative balance among major thrombus components may be more closely associated with the completeness of reperfusion than with procedural success or efficiency.

Previous studies have generally reported favorable endovascular responses in association with higher RBC content or lower platelet/fibrin content. Higher RBC proportions have been associated with successful reperfusion and fewer thrombectomy attempts, and meta-analytic evidence has linked greater RBC content to favorable angiographic outcomes [4,6,7,10,17]. Of particular relevance to our findings, platelet-rich thrombi have been associated with lower rates of complete reperfusion [9,18]. Nevertheless, associations between individual thrombus components and thrombectomy outcomes have not been consistent across studies [8,19]. This heterogeneity may partly reflect differences in histological methods, thrombectomy techniques, and definitions of component predominance, as well as the difficulty of representing a multicomponent thrombus using individual component proportions. Furthermore, no standardized criterion has been established for defining RBC-, platelet-, or fibrin-dominant thrombi [2].

The observed association is biologically plausible given the composition-dependent mechanical properties of thrombi. RBC-rich thrombi are generally softer and more deformable, allowing greater penetration and integration of stent struts, whereas platelet- and fibrin-rich thrombi tend to be stiffer and more resistant to deformation [2,4,5,20,21]. Fibrin-rich thrombi may additionally exhibit greater friction against the vessel wall, potentially increasing resistance during retrieval [22]. These properties may partly contribute to the observed association between greater relative RBC predominance and the completeness of final reperfusion. Nevertheless, final reperfusion is influenced by multiple thrombus-related and procedural factors, and thrombus mechanics themselves are determined not only by bulk composition but also by fibrin architecture, clot contraction, spatial heterogeneity, and other cellular and molecular constituents [5,23,24]. Thus, compositional balance should be regarded as a summary representation of one aspect of thrombus phenotype rather than a direct measure of its mechanical behavior.

An important feature of the present study is that thrombus composition was evaluated as a relative balance rather than as independent component percentages or dichotomous component-rich categories. Because compositional data are inherently characterized by the relationships among their constituent parts, log-ratio transformations provide a means of representing these relationships rather than considering each component in isolation [25]. An ILR-derived balance may therefore provide a complementary representation of the relative predominance of RBCs over platelets and fibrin without requiring dichotomization into component-rich or component-poor categories. This approach does not replace conventional analysis of individual thrombus components, but may provide an integrated perspective on their relative contributions. The present findings therefore extend previous observations linking RBC-rich or platelet/fibrin-poor thrombi with favorable reperfusion by suggesting that the RBC-to-platelet/fibrin balance itself may be relevant to complete reperfusion. Because thrombus composition is determined only after retrieval, however, the present compositional balance has limited immediate clinical applicability, and its current relevance is primarily mechanistic and hypothesis-generating.

The pattern of association also warrants cautious interpretation. Although compositional balance was significantly associated with complete reperfusion as a continuous variable, the effect estimate was modest and its confidence interval approached the null. Moreover, complete reperfusion increased from Q1 through Q3 with little further increase in Q4, and the difference between Q2 and Q1 was not significant. Thus, the data do not demonstrate a strict linear dose-response relationship. Accordingly, the continuous and ordinal-quartile effect estimates should be interpreted primarily as summary measures of the overall association rather than as evidence of a uniform linear dose-response relationship, while the observed quartile-specific pattern should be regarded as descriptive. The association appeared more evident in the upper compositional-balance quartiles, but this pattern should not be interpreted as evidence of a specific biological threshold and requires confirmation in an independent cohort.

The absence of an association with successful reperfusion should also be considered in the context of the high overall success rate. Successful reperfusion was achieved in 93.3% of patients, leaving limited variation for detecting differences according to thrombus composition. Such a ceiling effect may partly explain why compositional balance was associated with complete reperfusion but not with the conventional mTICI ≥ 2b endpoint. In this setting, complete reperfusion may have been more sensitive to composition-related differences in thrombectomy response. Complete reperfusion and procedural efficiency may also reflect different aspects of endovascular response. First-pass effect and the number of thrombectomy passes are influenced by multiple procedural and anatomical factors, whereas complete reperfusion specifically reflects the absence of residual reperfusion deficits at the end of the procedure. Thus, the observed association should not be interpreted simply as indicating easier thrombus retrieval; rather, compositional balance may be more specifically related to the completeness of final reperfusion. This is consistent with the heterogeneous results of previous studies regarding thrombus composition and first-pass effect [8,26,27,28].

The sensitivity analysis further supported the robustness of the principal finding. Because near-zero platelet values could disproportionately increase the ILR-derived compositional balance, patients with platelet proportions < 1% were excluded and quartiles were redefined. The association with complete reperfusion persisted, with effect estimates comparable to or slightly greater than those in the primary analysis. This finding indicates that the observed association was unlikely to be driven solely by a small number of thrombi with extremely low platelet proportions.

Several limitations should be acknowledged. First, this was a retrospective, single-center study. In addition, complete reperfusion was not a prespecified primary endpoint, and further regression modeling was performed following the initial comparison of multiple endovascular outcomes. Multiple endovascular outcomes and alternative parameterizations of compositional balance were evaluated without adjustment for multiple comparisons, which may increase the possibility of chance findings. Accordingly, these analyses should be considered exploratory and hypothesis-generating, and the observed associations require confirmation in independent cohorts. Second, histological analysis was necessarily restricted to patients from whom thrombus material was successfully retrieved, resulting in an inherent selection process in the study population. Because the availability of retrieved thrombus material may itself be related to clot characteristics, procedural factors, and treatment response, the analyzed thrombi may represent a selected subset of those encountered during EVT. This limitation should be considered when interpreting the present findings, which may not be generalizable to patients in whom thrombus material was not retrieved, particularly those with unretrieved or treatment-resistant thrombi. Third, the RBC, platelet, and fibrin proportions measured in this study represent only a subcomposition of the thrombus’s full biological composition and were not intended to constitute an exhaustive or mutually exclusive representation of all thrombus constituents. The compositional balance should therefore be interpreted specifically as the relative predominance among these three mechanically relevant components; other cellular and extracellular constituents were not captured by this measure. Fourth, retrieved thrombus material was not distinguished according to individual thrombectomy passes or retrieval sequence. In addition, histological assessment was necessarily based on sections obtained from the retrieved thrombus material and therefore could not fully capture the spatial organization or intrathrombus heterogeneity of the original thrombus. Accordingly, the measured component proportions should be interpreted as bulk compositional estimates of the retrieved thrombus material rather than as a complete representation of its spatially heterogeneous architecture, which may independently influence thrombectomy response [23,24]. Finally, the present study was focused on endovascular outcomes and did not evaluate whether the observed association between compositional balance and complete reperfusion translates into improved functional outcomes. Future studies integrating thrombus composition, procedural reperfusion characteristics, and clinical outcomes are warranted to determine whether the angiographic advantage associated with compositional balance ultimately translates into meaningful clinical benefit.

## 5. Conclusions

Higher thrombus compositional balance, reflecting greater relative RBC predominance over platelets and fibrin, was associated with a higher likelihood of complete reperfusion after mechanical thrombectomy, with a similar association observed in the sensitivity analysis. These exploratory findings suggest that the relative balance among thrombus components may provide additional insight into compositional factors associated with the completeness of endovascular reperfusion and warrant confirmation in independent cohorts.

## Figures and Tables

**Table 1:** Baseline characteristics of the study population.

Characteristics	Total (*n* = 312)
Age, years	71.2 ± 12.5
Male	150 (48.1)
Hypertension	223 (71.5)
Diabetes mellitus	98 (31.4)
Dyslipidemia	96 (30.8)
Current smoking	41 (13.1)
Atrial fibrillation	199 (63.8)
Initial NIHSS score	14 (10–19)
Stroke etiology	
Large-artery atherosclerosis	20 (6.4)
Cardioembolism	198 (63.5)
Undetermined etiology	94 (30.1)
Intravenous alteplase administration	119 (38.1)
Occlusion site	
Internal carotid artery	87 (27.9)
Middle cerebral artery	
M1 segment	114 (36.5)
M2 segment	76 (24.4)
Basilar artery	26 (8.3)
Other	9 (2.9)
Tandem occlusion	21 (6.7)
Onset-to-puncture time, minutes	249 (139–545)
First-line thrombectomy strategy	
Stent retriever	248 (79.5)
Contact aspiration	64 (20.5)
Thrombus composition	
Individual components	
RBC, %	36.6 (22.0–47.4)
Platelet, %	9.1 (3.8–17.9)
Fibrin, %	32.4 (21.5–47.4)
Compositional balance ^a^	0.62 (0.08–1.04)

Values are presented as mean ± standard deviation, median (interquartile range), or number (%), as appropriate. ^a^ Compositional balance represents the RBC-to-platelet/fibrin balance derived using an isometric log-ratio transformation. NIHSS, National Institutes of Health Stroke Scale; RBC, red blood cell.

**Table 2:** Comparison of endovascular outcomes across quartiles of thrombus compositional balance.

Endovascular Outcomes	Total (*n* = 312)	Q1 (*n* = 78)	Q2 (*n* = 78)	Q3 (*n* = 78)	Q4 (*n* = 78)	*p* Value ^a^	*p* for Trend ^b^
Successful reperfusion (mTICI ≥ 2b)	291 (93.3)	72 (92.3)	73 (93.6)	74 (94.9)	72 (92.3)	0.905	0.920
Complete reperfusion (mTICI 3)	205 (65.7)	44 (56.4)	49 (62.8)	57 (73.1)	55 (70.5)	0.114	0.030
First-pass effect	127 (40.7)	34 (43.6)	33 (42.3)	28 (35.9)	32 (41.0)	0.777	0.571
Number of passes	2 (1–3)	2 (1–3)	2 (1–3)	2 (1–3)	2 (1–3)	0.853	—
≥3 passes	92 (29.5)	23 (29.5)	21 (26.9)	25 (32.1)	23 (29.5)	0.920	0.824

Values are presented as median (interquartile range) or number (%), as appropriate. Compositional balance represents the RBC-to-platelet/fibrin balance derived using an isometric log-ratio transformation. ^a^ *p* values represent overall comparisons across the four quartiles. ^b^ *p* for trend was calculated for binary outcomes by treating quartiles of compositional balance as an ordinal variable. mTICI, modified Thrombolysis in Cerebral Infarction.

**Table 3:** Multivariable analysis of the association between thrombus compositional balance and complete reperfusion.

	Primary Cohort (*n* = 312)		Sensitivity Cohort (*n* = 299)	
Compositional Balance	Adjusted OR (95% CI)	*p* Value	Adjusted OR (95% CI)	*p* Value
As a continuous variable				
Per 1-unit increase	1.31 (1.03–1.67)	0.027	1.32 (1.03–1.68)	0.030
As quartiles				
Q1	Reference		Reference	
Q2	1.11 (0.55–2.27)	0.770	1.11 (0.54–2.29)	0.769
Q3	2.59 (1.22–5.49)	0.013	2.71 (1.25–5.88)	0.011
Q4	2.44 (1.15–5.17)	0.020	2.89 (1.34–6.23)	0.007
Per quartile increase	1.41 (1.11–1.80)	0.005	1.49 (1.17–1.91)	0.002

Adjusted ORs were estimated using binary logistic regression with complete reperfusion (mTICI 3) as the dependent variable. Models were adjusted for age, hypertension, diabetes mellitus, dyslipidemia, onset-to-puncture time, stroke etiology (large-artery atherosclerosis, cardioembolism, and undetermined etiology), occlusion site (internal carotid artery, M1 segment of middle cerebral artery, and other sites), intravenous alteplase administration, tandem occlusion, and first-line thrombectomy strategy (stent retriever or contact aspiration). The sensitivity cohort excluded 13 patients with a platelet proportion < 1%, and compositional-balance quartiles were redefined among the remaining 299 patients as Q1, ≤0.041; Q2, >0.041 to ≤0.596; Q3, >0.596 to ≤1.006; and Q4, >1.006. CI, confidence interval; mTICI, modified Thrombolysis in Cerebral Infarction; OR, odds ratio.

## Data Availability

The data presented in this study are available on request from the corresponding author.

## Supplementary Materials

The following supporting information can be downloaded at: https://www.mdpi.com/article/10.3390/jcm15208061/s1, Table S1: Baseline and procedural characteristics according to quartiles of compositional balance; Table S2: Univariable and multivariable logistic regression analyses for complete reperfusion in the primary cohort; Supplementary methods: R codes for calculating the compositional balance and for principal multivariable regression model.
