# Supplementary material for: Thrombus Compositional Balance Is Associated with Complete Reperfusion After Mechanical Thrombectomy
Source: J Clin Med. 2026 Oct 17;15(20):8061. doi: 10.3390/jcm15208061 (PMC13706571; doi:10.3390/jcm15208061)
Supplement: Supplementary file 1 [file jcm-15-08061-s001.zip › jcm-4594815-supplementary.pdf]

Supplementary Materials

Supplementary Tables

Table S1. Baseline and procedural characteristics according to quartiles of compositional balance

Characteristics	Q1 (*n* = 78)	Q2 (*n* = 78)	Q3 (*n* = 78)	Q4 (*n* = 78)	*P* value
Age, years	67.7 ± 14.3	72.7 ± 11.7	71.7 ± 11.2	72.6 ± 12.0	0.040
Male	32 (41.0)	45 (57.7)	36 (46.2)	37 (47.4)	0.206
Hypertension	54 (69.2)	62 (79.5)	57 (73.1)	50 (64.1)	0.185
Diabetes mellitus	22 (28.2)	36 (46.2)	25 (32.1)	15 (19.2)	0.003
Dyslipidemia	27 (34.6)	26 (33.3)	20 (25.6)	23 (29.5)	0.614
Current smoking	11 (14.1)	11 (14.1)	12 (15.4)	7 (9.0)	0.647
Atrial fibrillation	33 (42.3)	49 (62.8)	57 (73.1)	60 (76.9)	<0.001
Initial NIHSS score	12 (10–16)	14 (9–19)	15 (11–18.8)	13.5 (9–20)	0.339
Stroke etiology					<0.001
Large-artery atherosclerosis	2 (2.6)	9 (11.5)	4 (5.1)	5 (6.4)	
Cardioembolism	33 (42.3)	49 (62.8)	56 (71.8)	60 (76.9)	
Undetermined etiology	43 (55.1)	20 (25.6)	18 (23.1)	13 (16.7)	
Intravenous alteplase administration	22 (28.2)	36 (46.2)	34 (43.6)	27 (34.6)	0.079
Occlusion site					<0.001
Internal carotid artery	13 (16.7)	14 (17.9)	29 (37.2)	31 (39.7)	
Middle cerebral artery					
M1 segment	31 (39.7)	22 (28.2)	32 (41.0)	29 (37.2)	
M2 segment	25 (32.1)	33 (42.3)	7 (9.0)	11 (14.1)	
Basilar artery	6 (7.7)	7 (9.0)	8 (10.3)	5 (6.4)	
Other	3 (3.8)	2 (2.6)	2 (2.6)	2 (2.6)	
Tandem occlusion	2 (2.6)	9 (11.5)	5 (6.4)	5 (6.4)	0.168
Onset-to-puncture time, minutes	228 (135–430)	232 (139–519)	253 (145–559)	329 (142–639)	0.319
First-line thrombectomy strategy					0.053
Stent retriever	64 (82.1)	69 (88.5)	59 (75.6)	56 (71.8)	
Contact aspiration	14 (17.9)	9 (11.5)	19 (24.4)	22 (28.2)	

Compositional-balance quartiles were defined as Q1, ≤0.084; Q2, >0.084 to ≤0.624; Q3, >0.624 to ≤1.041; and Q4, >1.041. Values are presented as mean ± standard deviation, median (interquartile range), or number (%), as appropriate. *P* values were calculated using one-way analysis of variance for normally distributed continuous variables, the Kruskal–Wallis test for non-normally distributed continuous variables, and the Pearson chi-square test or Fisher's exact test, as appropriate, for categorical variables. NIHSS, National Institutes of Health Stroke Scale.

Table S2. Univariable and multivariable logistic regression analyses for complete reperfusion in the primary cohort

Variable	Unadjusted OR (95% CI)	*P* value	Adjusted OR (95% CI)	*P* value
Covariates				
Age, per 1-year	1.02 (1.00–1.04)	0.073	1.02 (0.99–1.04)	0.196
Hypertension	1.56 (0.94–2.58)	0.088	1.18 (0.65–2.15)	0.586
Diabetes mellitus	1.57 (0.93–2.65)	0.091	1.39 (0.79–2.46)	0.253
Dyslipidemia	2.02 (1.17–3.48)	0.011	1.99 (1.13–3.51)	0.018
Stroke etiology				
Large-artery atherosclerosis	Reference		Reference	
Cardioembolism	0.47 (0.15–1.45)	0.189	0.39 (0.12–1.26)	0.115
Undetermined etiology	0.44 (0.14–1.43)	0.172	0.64 (0.18–2.19)	0.473
Intravenous alteplase administration	1.43 (0.87–2.33)	0.154	1.15 (0.66–2.00)	0.624
Occlusion site				
Internal carotid artery	Reference		Reference	
M1 segment	0.80 (0.44–1.43)	0.442	0.99 (0.50–1.94)	0.971
Other sites	1.13 (0.62–2.07)	0.685	1.40 (0.70–2.81)	0.347
Tandem occlusion	2.33 (0.76–7.10)	0.137	2.21 (0.54–6.94)	0.113
Onset-to-puncture time, per 60-min	0.97 (0.94–1.00)	0.039	0.96 (0.93–1.00)	0.047
First-line thrombectomy strategy				
Stent retriever	Reference		Reference	
Contact aspiration	1.19 (0.66–2.14)	0.565	1.34 (0.67–2.67)	0.414
Compositional balance				
As a continuous variable				
Per 1-unit increase	1.23 (1.00–1.50)	0.050	1.31 (1.03–1.67)	0.027
As quartiles				
Q1	Reference		Reference	
Q2	1.31 (0.69–2.48)	0.415	1.11 (0.55–2.27)	0.770
Q3	2.10 (1.07–4.10)	0.031	2.59 (1.22–5.49)	0.013
Q4	1.85 (0.95–3.58)	0.069	2.44 (1.15–5.17)	0.020
Per quartile increase	1.27 (1.02–1.56)	0.030	1.41 (1.11–1.80)	0.005

Values are ORs with 95% CIs. Complete reperfusion (mTICI 3) was the dependent variable. For compositional balance, unadjusted estimates are shown for the continuous, categorical-quartile, and ordinal-quartile parameterizations; adjusted estimates correspond to those reported in Table 3. Compositional-balance quartiles were defined as Q1, ≤0.084; Q2, >0.084 to ≤0.624; Q3, >0.624 to ≤1.041; and Q4, >1.041. Adjusted estimates for individual covariates are from the multivariable model in which compositional balance was entered as a continuous variable. For regression analysis, occlusion site was categorized as internal carotid artery, M1 segment, and other sites; the latter comprised M2 segment, basilar artery, and all remaining occlusion sites. CI, confidence interval; mTICI, modified Thrombolysis in Cerebral Infarction; OR, odds ratio.

Supplementary Methods

(1) R code for calculating the compositional balance

```
# Calculation of the ILR-derived compositional balance

# Component proportions
data$rbc_pct      <- data$glya_stain / data$glya_total
data$platelet_pct <- data$cd42b_stain / data$cd42b_total
data$fibrin_pct   <- data$fib_stain / data$fib_total

# ILR balance: RBC versus platelet/fibrin
data$compositional_balance <-
  sqrt(2/3) * log(data$rbc_pct / sqrt(data$platelet_pct * data$fibrin_pct))
```

(2) R code for principal multivariable regression model

```
# Multivariable logistic regression with compositional balance
model_continuous <- glm(
  complete_reperfusion ~ compositional_balance +
    age + htn + dm + dyslipidemia +
    onset_to_puncture + stroke_etiology + occlusion_site +
    iv_thrombolysis + tandem_occlusion + first_line_strategy,
  data = data,
  family = binomial(link = "logit")
)

# Odds ratios and 95% confidence intervals
exp(cbind(
  OR = coef(model_continuous),
  confint.default(model_continuous)
))
```
